# Assessing the mental health literacy of healthcare workers at a Johannesburg tertiary hospital

**DOI:** 10.4102/sajpsychiatry.v31i0.2352

**Published:** 2025-01-30

**Authors:** Carla A. Smit, Belinda S. Marais

**Affiliations:** 1 Department of Psychiatry, Faculty of Health Sciences, University of the Witwatersrand, Johannesburg, South Africa

**Keywords:** mental health literacy, psychiatry, mental illness, mental health, Mental Health Literacy Scale, stigma, South African healthcare workers, developing country

## Abstract

**Background:**

Good mental health literacy (MHL) has proven to aid in providing adequate and timely care, promote positive attitudes towards mental health and assist in the integration of mental healthcare with other services. Studies have shown that enhancing the MHL of healthcare workers (HCWs) can help alleviate the burden of mental illness.

**Aim:**

The study aims to explore the MHL of HCWs at a tertiary hospital in Johannesburg.

**Setting:**

The study was conducted at Helen Joseph Hospital.

**Methods:**

A quantitative, descriptive, cross-sectional study via a self-administered questionnaire consisting of: (1) a demographic, work and exposure to mental illness and mental healthcare services questionnaire and (2) the Mental Health Literacy Scale (MHLS) was employed. The MHLS identifies people with low MHL who could benefit from further interventions.

**Results:**

Two hundred and fifty-two HCWs participated in the study. The overall median MHLS score was 129, in keeping with a previous study conducted in SA and Zambia. Younger HCWs with less than 5 years of experience scored higher. Among the various professions, doctors scored highest and nurses lowest. The anaesthetic and psychiatric departments obtained the highest MHLS scores. Personal exposure to mental illness and mental health services was associated with higher MHLS scores.

**Conclusion:**

This study highlighted areas where mental health awareness and education are lacking, which are crucial for improving MHL. Targeted interventions to fill these identified gaps are therefore recommended.

**Contribution:**

To our knowledge, this was the first South African study to assess MHL among tertiary-level HCWs across various professions.

## Introduction

Mental health literacy (MHL) is defined as ‘knowledge and beliefs about mental disorders which aid their recognition, management or prevention’.^1^ It consists of one’s ability to identify psychological distress or specific mental disorders, knowledge and beliefs around risks and causes of mental disorders, self-help interventions, available professional help, how to find information on mental health and attitudes that enable recognition and facilitate appropriate help-seeking.^1^ Good MHL can facilitate the creation of systems to prevent disease and enable timely diagnosis and treatment by identifying symptoms early, and it encourages positive attitudes towards mental health and help-seeking behaviour.^2,3,4^ It helps to integrate mental healthcare with other healthcare services, making it an essential predictor of favourable mental health outcomes.^3,5^ Conversely, low levels of MHL are related to delayed mental healthcare seeking or no treatment at all, increasing the risk of long-term adverse outcomes.^2,6^ Furthermore, it has been shown that improving the MHL of healthcare workers (HCWs) can reduce the burden of mental illness.^7^ Poor MHL among HCWs perpetuates stigma and results in inadequate management of those requiring treatment.^7^

Globally, MHL remains poor, especially in developing countries.^8^ International studies of HCWs found low awareness, stigmatising attitudes and incorrect beliefs about mental healthcare users (MHCUs).^9^ A study among Kenyan primary HCWs found low levels of diagnostic accuracy in their assessment of mental disorders.^5^ A systematic review published in the Arab Gulf region revealed that physicians and nurses had negative attitudes and insufficient knowledge and confidence in treating mental illness.^9^ Studies in China and Taiwan had similar findings, often resulting in deficient care for MHCUs from primary HCWs.^10,11^ An Australian study supported the theory that improved knowledge of and positive attitudes towards mental health were associated with better MHL, and found that psychiatrists and nurses had higher MHL than lay people.^12^ Nevertheless, studies regarding MHL are limited, and even more so in non-Western countries.^13^ A review of research on MHL in non-western countries reported significant gaps.^8^ Research regarding MHL has included various approaches, such as assessing factors influencing MHL, evaluating programmes that address MHL and measuring the MHL of different populations.^2^ However the measurements used have not been consistent and have thus failed to fully capture the breadth of MHL.^2^ Furthermore, most studies in developing countries were also vignette-based and lacked a scale-based scoring system.^12^

Mental illness is a significant contributor to South Africa’s (SA) burden of disease. According to the South African Stress and Health study, approximately a sixth of the population experience a mental illness per year.^14^ However, mental healthcare in SA remains vastly underfunded and under-supported, with only a fraction of the budget spent on mental health.^15^ According to a 2019 study of the South African psychiatrist workforce, per 100 000 population, there were 1.53 psychiatrists, with only 0.03 psychiatrists per 100 000 population in rural areas.^14,16^ As a result, three out of four people with mental illness in SA do not receive adequate care, with only 28% of adults with moderate to severe mental illness receiving treatment.^14,16^ This is further compounded by factors such as crime, gender-based violence, difficult economic circumstances and human immunodeficiency virus (HIV) or acquired immunodeficiency syndrome (AIDS).^14^ Despite the urgent call for improved mental healthcare and the positive impact of adequate MHL, limited studies have been conducted in SA to assess the MHL of HCWs and how to improve it. A study investigating the knowledge of and attitudes towards mental illness of nurses at clinics in the Western Cape revealed that 94% were unable to diagnose mental illnesses and had subtle negative attitudes and incorrect beliefs about psychotropic drugs.^8^ Another study at primary health facilities in SA and Zambia stated that implementing interventions to improve MHL in primary healthcare settings would improve competence and assist in bridging the treatment gap.^17^ However, studies regarding MHL have yet to be conducted in tertiary hospitals in SA and among all categories of HCWs. There is a need to understand the MHL of all HCWs as patients might present to any department or category of HCW, and all HCWs play a role in screening and identifying patients with mental illness.^11^

### Aim and objectives

This study aimed to explore the MHL of HCWs at a tertiary hospital in Johannesburg.

The study objectives were to:

1. Describe the sample of HCWs in terms of demographics, work characteristics and personal exposure to mental illness and mental healthcare services.
2. Assess the MHL of the sample, specifically: HCWs’ ability to recognise common mental disorders and psychological distress, their knowledge of the causation and risk factors of mental illness, self-treatment, professional help available, how to seek mental health information and attitudes of the participants towards mental illness, in particular those that promote recognition and appropriate help-seeking behaviour.
3. Determine whether any associations exist between HCW characteristics and overall MHL.

The authors hypothesised that HCWs would have low MHL levels, with higher scores among those with greater personal or professional exposure to mental health services and conditions.

## Research methods and design

### Study design and setting

This was a quantitative, descriptive, cross-sectional study, conducted at Helen Joseph Hospital (HJH). Helen Joseph Hospital is a tertiary-level, public sector general hospital, with an emergency department, medical, surgical and orthopaedic wards, a psychiatric unit, an ICU and high-care unit. It is also an academic teaching hospital of the University of the Witwatersrand.

### Study population and sampling strategy

The study population consisted of HCWs from the various departments at HJH, specifically doctors, nurses, allied and other health professionals trained to provide healthcare to patients. Inclusion criteria required participants to be HCWs employed at HJH who provided informed consent for the study. Data were collected from May to August 2023. Participants were recruited through convenience sampling – by word of mouth and individual outreach. Department heads and secretaries helped coordinate convenient times for questionnaire distribution. All data were securely managed and entered into Excel by the primary researcher.

According to the Human Resource Department at HJH, the number of HCWs employed during August 2022 was 1619: 339 medical doctors, 1092 nurses and 188 allied staff members. The sample size calculation for this study was based on the Mental Health Literacy Scale (MHLS) scores of primary HCWs in SA and Zambia obtained in a previous study.^17^ The equation used was:

To adjust for other factors, such as age, profession, years of experience and personal exposure, the sample size was increased by 10% per variable. The calculated minimum sample size was thus 240.

### Data collection and instrument

Data were collected using a self-administered questionnaire consisting of two sections. The first section included a demographic, work and exposure to mental illness and mental healthcare services questionnaire. The second part comprised the MHLS. The MHLS is a tool that assists in identifying people with low MHL who could benefit from further interventions.^7,12,18,19^ It is easy to administer, time-efficient, straightforward to score and methodologically robust.^12^

The MHLS consists of 35 items, covering the six attributes of MHL, namely:

1. Recognising disorders, including specific disorders and certain features or categories.
2. Knowledge of causation and risk factors, including social, genetic, biological and psychological.
3. Knowledge of self-treatment, including common treatments recommended by mental health professionals and activities that an individual can perform.
4. Knowledge of professional help available.
5. Knowledge of where to seek information and having the capacity to do so.
6. Attitudes that promote recognition or appropriate help-seeking behaviour: assessing negative attitudes, stigma and the willingness to engage in help-seeking behaviour.^3^

The total score for the MHLS ranges from 35 to 160 and is derived from summing all items, with a higher total score indicating better MHL.^19^ The first 15 questions use a 4-point Likert scale, and the last 20 use a 5-point Likert scale. Studies show that the MHLS has methodological advantages compared to other scale-based measures of MHL and has good validity and internal and test–retest reliability.^12^ A systematic review assessing the methodological quality of measurement tools found that the MHLS ranked highly on content validity, reliability and internal consistency.^18^ A study conducted in SA and Zambia found that it possesses strong content validity in low- and middle-income settings.^7^ The MHLS was developed by Dr O’Connor in 2015, initially created and tested in Australia, and is freely available.^7,12,18,19^ Dr O’Connor was informed of this study and permitted the change of questions 9 and 10, relating to Australia, to fit the South African context. Additionally, he suggested that questions 5 and 6 should be slightly modified, given the changes in the diagnostic and statistical manual of mental disorders, fifth edition (DSM 5).

Printed hardcopies of the questionnaire were distributed by the primary researcher, who explained the study to potential participants. Healthcare workers were additionally given an information sheet describing the research and its purpose. Completed questionnaires were collected using a sealed collection box to maintain anonymity. At every point of handling the data, steps were taken to maintain the data’s quality, integrity and reliability.

### Data analysis

The data were captured in Microsoft Excel^TM^. All statistical analyses were conducted using R software (R version 4.0.1; https://www.r-project.org). The data set was assessed for departure from normality using the Shapiro–Wilk tests and analysed using non-parametric statistics. Analyses were two-tailed, and the model-level significance was set at 0.05. To analyse the relationship between demographic and work characteristics against the six attributes and the total score on the MHLS, Mann–Whitney U tests were used for variables with two factors and Kruskal–Wallis tests for more than two factors, followed by pairwise Mann–Whitney U tests for specific outcomes.

### Ethical considerations

Approval was obtained from the University of the Witwatersrand Human Research Ethics Committee (clearance number M230243 MED23-01-104) and the HJH Research Committee and Chief Executive Officer. Study participation was voluntary, anonymous and confidential. Because of it being a self-administered questionnaire, consent was implied by the completion and return. A distress protocol was provided at the end of the survey.

## Results

### Objective 1: Participants

**FIGURE 1:**
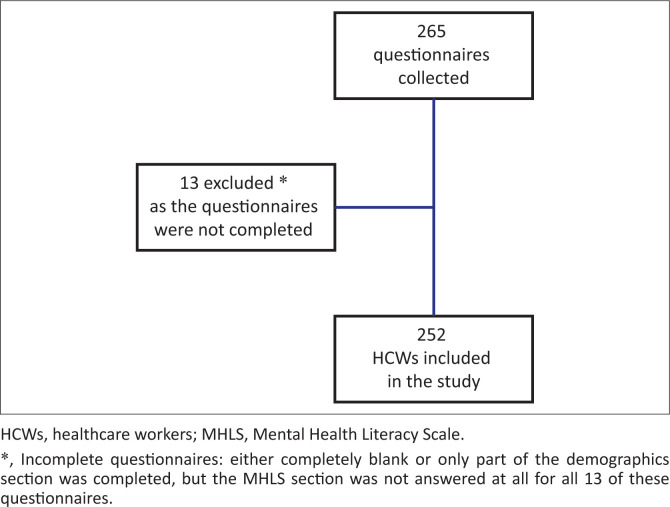
Flow diagram of healthcare workers participating in the study.

Two hundred and fifty-two HCWs at HJH were included in this study (Figure 1). The median age of participants was 32 (IQR [interquartile range] 13.25). The demographics, work characteristics and personal exposure to mental illness and mental healthcare services of the HCWs are shown in Table 1.

**TABLE 1:** Demographics, work characteristics and personal exposure to mental illness and mental healthcare services of the healthcare worker study participants.

Variables	*n*	%
**Age (years)**		
20–30	109	43.3
31–40	74	29.4
41–50	47	18.7
> 50	22	8.7
**Gender**		
Female	195	77.4
Male	57	22.6
**Race**		
Black people	150	59.5
White people	47	18.7
Indian people	42	16.7
Mixed race people	12	4.8
Asian people	1	0.4
**Relationship status**		
Married	101	40.1
Single	76	30.2
In a relationship	63	25.0
Did not answer	12	4.8
**Profession**		
Doctor	105	41.7
Nurse	91	36.1
Allied	47	18.7
Other	9	3.6
**Years practising**		
0.5–5	108	42.9
6–10	68	27.0
> 10	76	30.2
**Department**		
Anaesthetics	20	7.9
Casualty	32	12.7
Internal medicine	28	11.1
Outpatients	25	9.9
Psychiatry	42	16.7
Surgical	53	21.0
Other	52	20.6
**Know someone with a mental illness**		
Yes	171	67.9
No	77	30.6
Did not answer	4	1.6
**Ever been diagnosed with mental illness**		
Yes	33	13.1
No	215	85.3
Did not answer	4	1.6
**Contact with mental health services**		
Yes	133	52.8
No	117	46.4
Did not answer	2	0.8

### Objective 2: Mental Health Literacy Scale scores

Scores for the six MHLS attributes and the total MHLS scores of HCWs who participated in the study are shown in Table 2.

**TABLE 2:** Mental Health Literacy Scale scores for the six attributes and total scores of the healthcare worker study participants.

MHLS scores	Median	IQR	IQR/median
Attribute 1: Ability to recognise disorders *(Q:1–8; S:8–32)*	28	25–30	0.18
Attribute 2: Knowledge of causation and risk factors *(Q:9–10; S:2–8)*	6	5–6	0.17
Attribute 3: Knowledge of self-treatment *(Q:11–12; S:2–8)*	6	5–7	0.33
Attribute 4: Knowledge of professional help available *(Q:13–15; S:3–12)*	10	8–11	0.30
Attribute 5: Knowledge of where to seek information *(Q:16–19; S:4–20)*	16	15–19	0.25
Attribute 6: The attributes to promote recognition or appropriate help-seeking behaviour *(Q:20–35; S:16–80)*	64	56–71	0.23
Total score *(Q:1–35; S:35–160)*	129	118–139	0.16

MHLS, Mental Health Literacy Scale; IQR, interquartile range; Q, MHLS question numbers; S, minimum and maximum possible scores for those questions.

### Objective 3: Association between healthcare worker characteristics and total Mental Health Literacy Scale scores

The association between demographic and work characteristics of the participants and the total MHLS scores is shown in Table 3. The association between personal exposure to mental illness and mental health services and total MHLS scores is shown in Table 4.

**TABLE 3:** Association between healthcare workers’ demographic and work characteristics and total Mental Health Literacy Scale scores.

Variables	Mean	Range	Median	Statistics		
				KW	*df*	*p*
**Age (years)**	-	-	-	38.83	3	0.001
20–30	134.2	102–157	136.0	-	-	-
31–40	123.9	89–153	126.0	-	-	-
41–50	119.8	86–147	119.0	-	-	-
> 50	125.2	105–144	127.5	-	-	-
**Profession**	-	-	-	61.66	3	0.001
Doctor	134.3	104–155	135.0	-	-	-
Nurse	117.8	86–150	117.0	-	-	-
Allied	132.8	111–157	134.0	-	-	-
Other	124.7	98–140	128.0	-	-	-
**Department**	-	-	-	20.09	6	0.001
Anaesthetics	136.1	118–146	137.5	-	-	-
Casualty	122.4	98–151	122.0	-	-	-
Internal medicine	122.3	88–143	123.0	-	-	-
Outpatients	123.2	89–151	122.0	-	-	-
Psychiatry	134.2	94–155	136.0	-	-	-
Surgical	125.5	86–153	128.0	-	-	-
Other	129.9	98–157	130.0	-	-	-
**Number of years practising**	-	-	-	35.82	2	0.001
0–5	134.1	89–157	135.5	-	-	-
6–10	121.9	86–153	123.5	-	-	-
> 10	123.8	89–152	124.0	-	-	-

KW, Kruskal–Wallis test; *df*, degrees of freedom.

**TABLE 4:** Association between healthcare workers’ personal exposure to mental illness and mental health services and total Mental Health Literacy Scale scores.

Variables	Mean	Range	Median	Statistics	
				*U*	*p*
**Knowing someone with a mental illness**	-	-	-	3853	0.001
Yes	133.9	104–157	134	-	-
No	127.0	86–153	118	-	-
**Being diagnosed with mental illness**	-	-		2600.5	0.014
Yes	132.3	89–157	135	-	-
No	122.8	86–155	129	-	-
**Contact with mental health services**	-	-	-	4927.5	0.001
Yes	131.5	89–157	134	-	-
No	120.0	86–155	123	-	-

U, Mann–Whitney U tests.

## Discussion

### Participants

The majority of participants were in the 20–30 year old age group with 0–5 years of practising in their profession, most likely because of HJH being an academic training hospital, where many HCWs start their training and/or are busy pursuing their specialisation. Furthermore, the majority of HCWs were female, in keeping with a previous study of South African doctors which also found this demographic shift of an increasing number of females compared to males.^20^ Similarly, an Australian study conducted in 2021 showed that females comprised 76% of HCWs, including doctors, nurses and allied staff.^21^ Most participants in the current study were doctors, which could be because of selection bias as the primary researcher is a doctor, and therefore more doctors could have opted to participate. The highest proportion of participating HCWs were from the surgical department, which corresponds to this being a large department, followed by the ‘other’ departments (which may have pharmacists or allied HCWs who cover multiple departments) and then the psychiatry department, which is a smaller department in HJH. The latter finding might thus again indicate selection bias, as psychiatric staff may have been more open to conducting a survey in their line of work. Regarding personal exposure to mental illness, the majority of HCWs reported knowing someone with a mental illness, which is in keeping with a previous study on South African doctors.^20^ However, a minority of HCWs reported a personal diagnosis of a mental illness, which differs markedly from rates in the general population – locally and internationally. In the South African Stress and Health (SASH) study, the lifetime prevalence of mental illness was 30% and internationally it has been reported as 29%, which is more than double the rate reported in this study of HCWs.^14,22^ Multiple studies have also shown that mental illness is increasingly prevalent in HCWs because of a variety of compounding factors such as burnout, stress and compassion fatigue.^23^ Possible reasons for HCWs under-reporting their own mental illness include perceived stigma from colleagues, fear of being ostracised and over-reliance on self-treatment.^23^ Contact with mental health services was not explicitly defined for participants, but was included to assess their potential exposure to such services.

### Mental Health Literacy Scale scores

The overall MHLS scores in the current study (median 129) revealed moderate overall MHL with a broad range of responses with variability around the median (IQR 118–139). These results were similar to those found in a study on primary HCWs in SA and Zambia using the MHLS, with a mean of 122.^17^ In the current study, the scores of the different attributes of MHLS were similar overall, with neither the knowledge-related (attributes 1–5) nor attitude-related attributes (attribute 6) scoring markedly higher. In terms of variability around the median, the IQR/median ratio was calculated which showed the greatest variability of responses for the following attributes: knowledge-related attributes – knowledge of self-treatment (0.33), knowledge of professional help available (0.30) and knowledge of where to seek information (0.25); followed by the attitude-related attribute to promote recognition or appropriate help-seeking behaviour (0.23). Although not directly comparable, in the SA and Zambia study the co-efficient of variation (CV) was calculated, which is the standard deviation/mean ratio, and their findings were that there was a greater relative dispersion around the mean for the knowledge-related attributes, specifically: knowledge of causation and risk factors (CV 23% i.e. a ratio of 0.23), knowledge of self-treatment (0.23), knowledge of where to seek information (0.23).^17^

Studies show inadequate MHL levels across various countries. According to a study by Elyamani et al., HCWs worldwide hold stigmatising attitudes, lack awareness and have false beliefs about patients with mental disorders.^9^ Similarly, a study conducted in Singapore between 1995 and 2016 showed inadequate MHL levels.^13^ A review of HCWs in Arab Gulf countries also indicated limited knowledge, low awareness of common disorders, negative attitudes and a lack of confidence on mental health topics.^9^ Studies in China and the UAE had similar findings.^19,24,25^ Various studies have also shown that developed countries have higher MHL than developing regions as they have more budget and resources allocated towards mental health which may lead to improved knowledge, and better awareness.^19,26,27^

### Healthcare workers’ demographic and work characteristics and Mental Health Literacy Scale scores

In this study, younger HCWs had the highest overall MHLS scores, which is in keeping with international findings. A Chinese study regarding the MHL of non-mental HCWs found that younger age was associated with higher MHL.^11^ This was supported by a study conducted in Australia and New Zealand showing that HCWs over age 60 had significantly lower MHL.^6^ Similar results have also been found in the general population, with a systematic review of MHL in Singapore which revealed that younger people were more likely to recommend seeing a psychiatrist, were more open to seeking mental healthcare and had a better understanding of mental illnesses.^13^ In contrast though was a study on Turkish general HCWs that found only a weak positive correlation between age and MHL, possibly attributed to the participants’ age distribution and likely cultural characteristics.^28^ Possible reasons for the association between younger age and better MHL could be that younger HCWs have recently completed their studies and are thus more up-to-date with current mental health knowledge and information, while older generations could possibly be more conservative in their views and hold more rigid cultural beliefs regarding mental illness and consulting mental HCWs. Generation Z is more proactive in addressing their mental health issues, as per the American Psychological Association.^29^ They have higher rates of reporting mental health issues, attending therapy, and are more willing to pay for mental healthcare services.^29^

Healthcare workers with fewer years of practice in the current study had higher MHL than those more experienced in their fields, which corresponds with the study’s finding that younger HCWs scored better on the MHLS. In the literature, it has been found that more experience in the psychiatric field leads to higher MHL. In Singapore, nurses who had more than 10 years of psychiatric experience were found to have better attitudes towards mental illness, whereas, in the study on primary HCWs in SA and Zambia, nurses with less mental health exposure held negative perceptions and attitudes towards mental illness.^13,17^ In the current study, years of healthcare practice was assessed, not years of practice in mental health.

A significant association was found between profession and MHLS scores. Doctors scored the highest, followed by allied HCWs, with nurses having the lowest MHLS scores. Not many studies assessing the MHL of allied HCWs were found; however a review of MHL in Singapore found that allied HCWs had mental health knowledge similar to that of the nursing staff.^13^ It should be noted that the group of allied HCWs that participated in the current study included psychologists, who are trained in mental health, and this may have skewed the results. Regarding nursing staff scoring the lowest on the MHLS compared to other HCWs, this was in keeping with findings from several other studies. A review of literature in the Arab Gulf region revealed that nurses had negative attitudes and inadequate knowledge of mental illnesses, with the majority unable to identify common disorders.^9^ This was further supported by a study in Singapore, which found that only about a third of nursing staff recommended seeing a psychiatrist for mental health concerns, and their diagnostic ability was much lower than that of doctors.^13^ A study involving primary healthcare nurses in the Western Cape also found similar results, with the majority of participants having difficulty with recognising mental disorders, poor knowledge regarding mental illness, as well as some negative attitudes towards mental illness.^30^ A subsequent study, conducted in KwaZulu-Natal (KZN), showed that nurses had relatively positive attitudes towards psychiatric patients but lacked the knowledge to identify and manage them.^31^ Factors which may contribute to negative attitudes among South African nurses in particular may include high levels of burnout, being overworked, understaffed and high rates of workplace violence.^32,33^ It has been shown that a meaningful nurse–MHCU relationship can decrease the length of hospital stay, improve the quality of life and decrease the severity of symptoms experienced, which further motivates improving MHL among nurses^34^ and emphasises the importance of including a dedicated mental health subject in nursing undergraduate training in SA, otherwise the problem will worsen rather than improve.

In the current study, HCWs from the anaesthetics department had the highest MHLS, followed closely by HCWs from the psychiatric department. There is a lack of literature on the MHL of anaesthetists specifically; however, it should be noted that all the HCWs from the anaesthetics department who participated in this study were doctors, as opposed to any other profession, which may have positively skewed the results. Healthcare workers from the psychiatric department scoring higher than the other departments, was an anticipated finding, as these HCWs have chosen to work in mental health, thus likely have less stigmatising attitudes to mental illness, and their daily involvement in managing psychiatric patients would allow for more experience in identifying symptoms and knowledge regarding mental illness. Furthermore, this finding was in keeping with previous research. A New Zealand and Australian study revealed that HCWs who had worked with mentally ill patients scored higher on MHL scores, and a survey of Chinese nurses showed that having worked in a psychiatric hospital was a positive predictor of MHL.^6,19^ A Singaporean review found that psychiatric nurses could identify common symptoms more accurately than general staff and were more likely to refer patients to a mental health professional for treatment and that mental HCWs were more optimistic regarding treatment outcomes.^13^ The South African study of nurses in KZN showed that experience with psychiatric patients led to more positive attitudes.^31^ However, a study of South African doctors found that both psychiatric and non-psychiatric doctors held negative attitudes towards MHCUs, but the psychiatric doctors still scored better overall.^20^ Conversely, the finding that non-mental HCWs in the current study fell short on knowledge of and attitudes towards mental illness was also in keeping with the literature. Only a third of clinic doctors and half of the general practitioners in a Singaporean study were able to identify and diagnose schizophrenia and depression, and in China, just over half of non-psychiatric HCWs were able to identify common disorders.^11,13^ A study in the Arab Gulf region found that among general practitioners and primary care physicians, stigmatisation and shame were present in treating MHCUs, which was further corroborated by the study of South African and Zambian primary HCWs which found negative stereotypes towards mental illnesses.^9,17^

### Healthcare workers’ personal exposure to mental illness and mental health services and Mental Health Literacy Scale scores

This study found that HCWs who had personal exposure to mental illness, whether through knowing someone, their own diagnosis, or exposure to mental health services, had significantly higher MHLS scores as compared to those with no personal exposure. The South African study regarding doctors’ attitudes to mental illness and psychiatry supported the current study’s results, in that it was also found that personal exposure to mental illness reduced fear, improved communication with patients and led to more positive attitudes.^20^ A Turkish study found that nursing students with a family member with mental illness possessed more knowledge about accessing mental health information, showed less stigmatisation, had more empathy and were better able to cope with mental illness.^35^ Similarly, a study conducted in Australia and New Zealand showed that individuals who had a close friend or family member with a mental illness were more likely to recognise symptoms and have knowledge about treatments.^6^ The study also revealed that individuals who had experienced similar problems as those presented in vignettes had higher scores on some MHL scales.^6^ However, in contrast to these findings, the South African study conducted on primary healthcare nurses in KZN found no significant relationship between personal contact with mental illness and knowledge or attitudes towards it.^31^

### Strengths and limitations

Limitations to this study include the possibility of social desirability and selection bias. An additional limitation is that this study was conducted at a single hospital and the results should therefore be interpreted critically concerning generalisability. Lastly, it is essential to note that MHL is a broad concept, and the literature suggests that no single tool can capture its entirety.^18^ Strengths of this study include the fact that it provides valuable information on MHL which has received limited attention in developing countries.

### Recommendations

To improve attitudes towards, and knowledge of, mental illness, targeted campaigns and interventions are necessary. According to a recent systematic review, educational programmes targeted at HCWs can assist with improving overall MHL.^36^ The findings from the current study revealed that programmes should be directed towards older HCWs with longer years of practice. These HCWs are more experienced in specialised fields, but may not be up-to-date with current psychiatric knowledge; therefore, they could benefit from tailored training programmes and continuous learning interventions to update and maintain knowledge.^37^ Younger HCWs can also be utilised as a tool for change to promote knowledge, awareness and positive attitudes towards mental health by leading open discussions and combatting stigma.^38^ Differences in the level of MHL among HCWs can significantly impact the quality of care that patients receive. It is therefore essential to ensure that all HCWs have the necessary MHL. This can be achieved through targeted interventions to provide nursing staff – who scored the lowest MHL – training on causation, symptom identification and information-seeking, from including it in their undergraduate degree to continuous training. Non-psychiatrically trained departments should also be targeted for awareness and educational programmes. The findings can also be used as a baseline to plan interventions and measure their efficacy.

## Conclusion

There is a pressing global need for effective interventions to transform MHL. It is critical to not only equip more HCWs with adequate MHL but also to enhance the capabilities of the current workforce through targeted campaigns. Identifying gaps in education and awareness is essential to improving MHL and determining the most valuable and effective interventions. This study found that older participants with more years of experience, particularly in non-psychiatric departments and the nursing profession, had lower MHL. Therefore, educational programmes and interventions should be focused on these particular groups to improve MHL and ultimately help alleviate the burden of mental illness in SA.
